# Is the Impact of Sodium–Glucose Co-Transporter 2 (SGLT2) Inhibitors on Bone Metabolism and Fracture Incidence a Class or Drug Effect? A Narrative Review

**DOI:** 10.3390/medicines12020010

**Published:** 2025-04-16

**Authors:** George I. Lambrou, Athanasia Samartzi, Eugenia Vlachou, Athanasios N. Tsartsalis

**Affiliations:** 1 Choremeio Research Laboratory, First Department of Pediatrics, National and Kapodistrian University of Athens, Thivon & Levadeias Str. 8, 11527 Athens, Greece; glamprou@med.uoa.gr; 2 University Research Institute of Maternal and Child Health & Precision Medicine, National and Kapodistrian University of Athens, Thivon & Levadeias Str. 8, 11527 Athens, Greece; 3 Laboratory for the Research of the Musculoskeletal System “Th. Garofalidis”, Medical School, National and Kapodistrian University of Athens, Athinas Str. 10, 14561 Athens, Greece; 4 Department of Endocrinology Diabetes and Metabolism, Naval Hospital of Athens, Dinokratous 70, 11521 Athens, Greece; nasiasamartzi@gmail.com; 5 Department of Nursing, School of Health Sciences, University of West Attica, Ag. Spydironos 28, 12243 Athens, Greece; evlachou@uniwa.gr

**Keywords:** Diabetes Mellitus type-2, SGLT-2, SGLT-2 inhibitors, bone mineral density, bone metabolism and fractures

## Abstract

**Background/Objectives**: Type 2 diabetes mellitus (T2DM) has a growing prevalence, even in developed countries. Because of the increase in life expectancy, the number of older people with T2DM is also increasing. The management and handling of these patients is challenging due to its co-morbidities. **Aim**: In the present study, we reviewed the literature in order to investigate the impact of sodium–glucose co-transporter 2 inhibitors (SGLT-2 inhibitors) on bone metabolism and fracture incidence. Methods: We searched the literature using the databases of PubMed, CENTRAL and Cochrane Central Register of Controlled Trials up to December 2024. **Results**: There is a controversial position in the literature concerning the effects of SGLT2 inhibitors when administered in T2DM, with respect to bone metabolism and bone fracture incidence. Multiple studies suggest the SGLT2 inhibitors have a disadvantageous effect on bone metabolism and fracture incidence, while several others suggest a beneficial effect. **Conclusions**: Patients with type 2 diabetes mellitus are at high risk of alterations in their bone metabolism. SGLT2 inhibitors are a novel class with pleiotropic effects in many organs, such as the kidneys and heart, although their effect on bone metabolism and fracture incidence is still unclear. Until we have more clinical data, all caregivers (medical and nursing staff) should be aware of possible bone fractures in patients receiving this class of agents.

## 1. Introduction

*On Diabetes Mellitus*

Diabetes mellitus (DM) is a metabolic disorder that is one of the most common chronic diseases and one of the most important causes of premature mortality [1]. It is a chronic disease with social and economic implications that requires coordinated action in many sectors. DM is a global health problem and one of the leading causes of morbidity and mortality. Although the incidence of DM varies among populations due to differences in genetic susceptibility and other risk factors, the incidence of the disease is high worldwide [2]. DM is one of the most economically demanding chronic diseases worldwide. According to the International Diabetes Federation (IDF), 382 million people suffered from diabetes in 2013, and this number is expected to increase to 592 million by 2035, while 175 million people remain undiagnosed. In total, 90% of these people suffer from type 2 diabetes (T2DM) [3]. Similarly high levels have been recorded in Canada, where approximately 1.4 million people have already been affected by T2DM. DM no longer only affects older people but also younger people, and according to estimates in the USA, it has been observed in 1.5 million cases in people over the age of 20. Similarly, in Europe, there has been a significant increase in the incidence of type 2 diabetes, not only in the elderly population but also in people under 20–30 years of age, making diabetes a major public health problem [2,4,5]. Diabetes is considered to be Europe’s “silent pandemic”. In 2019, it was estimated that over 60 million Europeans suffered from diabetes, with the prediction of reaching 66 million by the year 2030 and more than 80 million by the year 2045 [4,5]. Thus, recent research indicated that patients with diabetes will probably account for a population corresponding to the second largest state in Europe, affecting more than 10% of Europe’s population.

The treatment of diabetes currently focuses on maintaining blood glucose levels at levels that are as close to normal as possible with medication, diet and exercise. The conservative treatment of diabetes with a healthy diet (diet, exercise) and medication (insulin and hypoglycemic tablets) is considered the cornerstone of the long-term treatment of the disease [6,7]. At the same time, continuous laboratory tests are performed to evaluate the treatment plan that is being implemented, and patients with diabetes are monitored by a team consisting of a doctor, a nurse and a number of other specialists, such as a dietitian, a pharmacist, a psychologist, a podiatrist and a neurologist. As the patient is called upon to take an active role, nursing care focuses on educating the individual to manage the disease. In this context, the health professional should provide accurate information to people with diabetes on self-management of medication, diet planning, exercise, self-assessment and self-care.

Thus, in the present, study we reviewed the literature in order to investigate the impact of sodium–glucose co-transporter 2 inhibitors (SGLT-2 inhibitors) on bone metabolism and fracture incidence. The relation between these two topics is still the subject of intensive investigation and research, as it represents a very important aspect of bone metabolic diseases and diabetes.

## 2. Literature Search

We searched the literature on the databases of PubMed, CENTRAL and Cochrane Central Register of Controlled Trials up to December 2024. A computerized search in the databases was accomplished by using Medical Subject Headings and entry terms such as diabetes mellitus type 2, SGLT2 inhibitors, bone mineral density (BMD), bone metabolism and fractures.

The studies included in the current systematic review were retrieved from an independent literature search performed in the PubMed and Cochrane databases, as well as other sources like Google Scholar. Independent keywords, along with their combinations, were applied to both databases. The specific keywords were the following: “diabetes mellitus type-2”, “SGLT-2”, “SGLT-2 inhibitors”, “bone mineral density”, “bone metabolism” and “fractures”. The aforementioned keywords were generated through an evaluation of the MeSH (Medical Subject Headings) database. Specifically, the search string that was applied to PubMed was as follows: ((((((diabetes mellitus type-2 [Title/Abstract]) OR T2DM [Title/Abstract]) AND SGLT-2 [Title/Abstract])) OR (((SGLT-2 inhibitors [Title/Abstract]) OR SGLT2 inhibitors [Title/Abstract]) AND bone mineral density [Title/Abstract]))) OR (((BMD [Title/Abstract]) OR bone metabolism [Title/Abstract]) AND fractures [Title/Abstract]). The search string that was applied to the Cochrane database was as follows: (Diabetes Mellitus Type-2 AND SGLT-2 Inhibitors OR Bone Mineral Density AND SGLT-2 Inhibitors OR SGLT-2 Inhibitors AND Fractures).

In order to be included in the current review, a study needed to adhere to specific inclusion and exclusion criteria. In particular, a study should have provided results regarding SGLT-2 inhibitors and bone metabolism. Articles for which the full text was not available were also excluded. Time or country of origin restrictions were not applied during the identification of eligible studies, whereas studies that were published in languages other than English were not considered eligible for inclusion.

## 3. Type 2 Diabetes Mellitus and Bone Metabolism

As mentioned above, type 2 diabetes mellitus (T2DM) has a growing prevalence, even in developed countries. Because of the increase in life expectancy, the number of older people with T2DM is also increasing. The management and handling of these patients is challenging due to its co-morbidities. Older patients may also suffer from cardiovascular events, diabetic nephropathy, diabetic retinopathy and osteoporosis [8]. Although it has been reported, especially in experimental studies, that there is an association between T2DM and osteoporosis, the exact mechanism remains unknown. It is believed that fractures are a consequence of the deterioration of the bone microenvironment and bone quality, rather than a decrease in bone mineral density (BMD) [9].

### 3.1. Bone Metabolism

**Figure 1:**
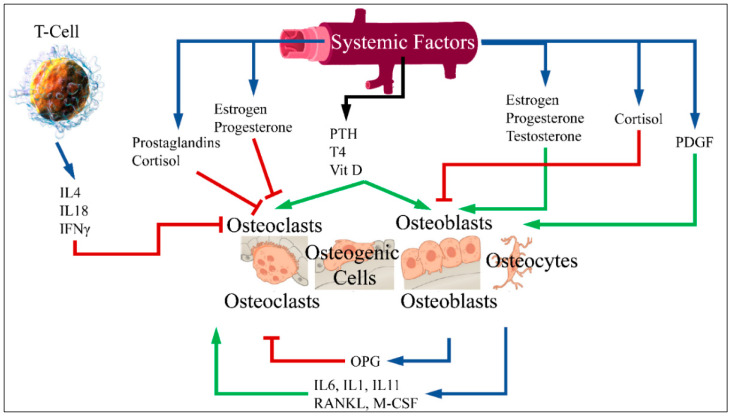
RANKL is an osteoclast differentiation factor. This factor binds to the RANK receptor in primary osteoclast cells and leads to the differentiation and activation of osteoclasts. RANK enhances the action of osteoclasts, while OPG binds to RANKL and therefore inhibits bone resorption. An imbalance in OPG/RANKL/RANK expression is responsible for osteoporosis [14] (legend: IL: Interleukin; IFNγ: Interferon gamma; PTH: Parathormone; T4: Thyroxine; Vit D: vitamin D; PDGF: Platelet-Derived Growth Factor; OPG: osteoprotegerin; RANKL: receptor activator of nuclear factor kappa-Β ligand; M-CSF: Macrophage colony-stimulating factor. The blue arrows imply production; red arrows imply inhibition; and green arrows imply stimulation) (figure adopted and reproduced from Daniilopoulou et al. (2022) [13] under the Creative Commons License CY 4.0 doi:10.3390/medicina58020224).

Bone metabolism is a continuous process throughout an individual’s life span, by which bones renew with resorption and growth. This process is guided by a dynamic relationship between osteoclasts, osteoblasts and hormonal regulation. An important role is held by the receptor activator of the nuclear factor-κB ligand (RANKL)/osteoprotegerin (OPG) pathway. RANKL is a cytokine belonging to the Tumor Necrosis Factor (TNF) family, which binds RANK in osteoclast precursors, promoting its differentiation to mature osteoclasts. On the other hand, RANKL binds to OPG. The latter is secreted from osteoblasts and inhibits osteoclast activity [10]. Bone formation is performed by stimulating osteoblasts and inhibiting osteoclasts. Bone resorption is performed by an active osteoclast and stimulated by RANKL. RANKL is a cytokine belonging to the Tumor Necrosis Factor (TNF) family. This factor connects to the RANK receptor in osteoclast progenitor cells and leads to the differentiation and activation of osteoclasts. Moreover, RANKL is bound by OPG and secreted by osteoblasts, which inhibits osteoclast differentiation [11,12,13], on which we have previously reported [13] (Figure 1).

### 3.2. Antidiabetic Treatments and Bone Metabolism

An impact on bone metabolism has been proven for the majority of antidiabetic agents, especially for thiazolidinedione (TZDs) and insulin [15]. The only approved representative of the class of TZDs, pioglitazone, is a synthetic peroxisome proliferator-activated receptor γ (PPARγ) stimulator, and it is a potent insulin sensitizer with multiple mechanisms of action. This agent increases glucose consumption in muscle tissues; reduces the levels of glycerol, free fatty acids and triglycerides; and increases insulin sensitivity in the muscles, adipose tissue and liver. In addition, it inhibits hepatic gluconeogenesis, promoting a decrease in the production of endogenous glucose [16]. On the other hand, basal insulin reduces high glucose levels through a reduction in hepatic glucose production and suppression of lipolysis. A previous study demonstrated that women with diabetes who were treated for a long period with TZDs had an increased incidence of fractures [17,18].

As for sulfonylureas, the findings from previous studies remain controversial. Most of them indicate that this class of drugs has raised the incidence of fractures, especially in older patients, probably because of a high risk of hypoglycemia and the risk of falling [19]. However, a recent meta-analysis demonstrated a reduced risk of fractures, although not statistically significant. The existing literature on Dipeptidyl Peptidase-4 Inhibitors (DPP-4 inhibitors) revealed neutral effects on the fracture incidence [20]. In contrast, Glucagon-Like Peptide-1 Receptor Agonists (GLP1RAs) had a beneficial effect on bone metabolism, although this could not be extended to a reduced fracture incidence [13].

**Figure 2:**
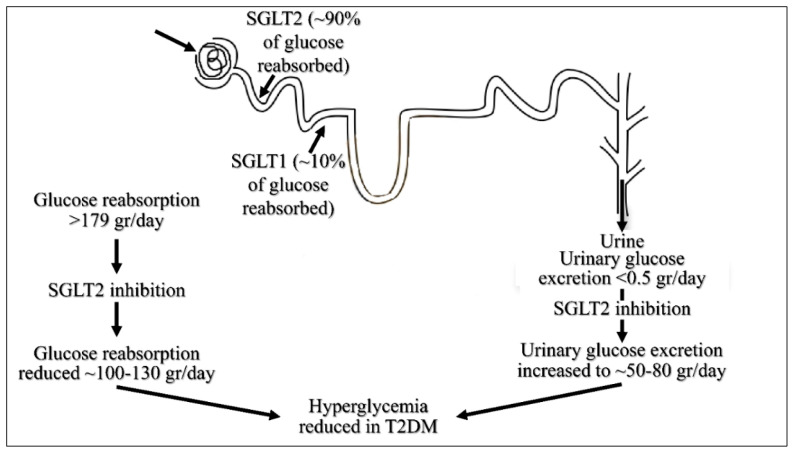
The mechanism of action of SGLT2 inhibitors, lowering plasma glucose (adopted and reproduced from By Lyfjaskvís’s own work, CC BY-SA 4.0, https://commons.wikimedia.org/w/index.php?curid=73298770, under the Creative Commons License CY 4.0, accessed 30 December 2024).

SGLT2 inhibitors are a class of antidiabetic agents, which reduce renal tubular glucose reabsorption in a glucose-dependent manner without the mediation of insulin release [21] (Figure 2). Although this class of inhibitors emerged in the last decade, their action and physiological machinery still remains to be elucidated. In addition, it is only recently that studies concerning other physiological systems such as the cardiovascular, renal and metabolism systems have emerged to investigate the inhibitors’ action in co-treatments [22].

**Figure 3:**
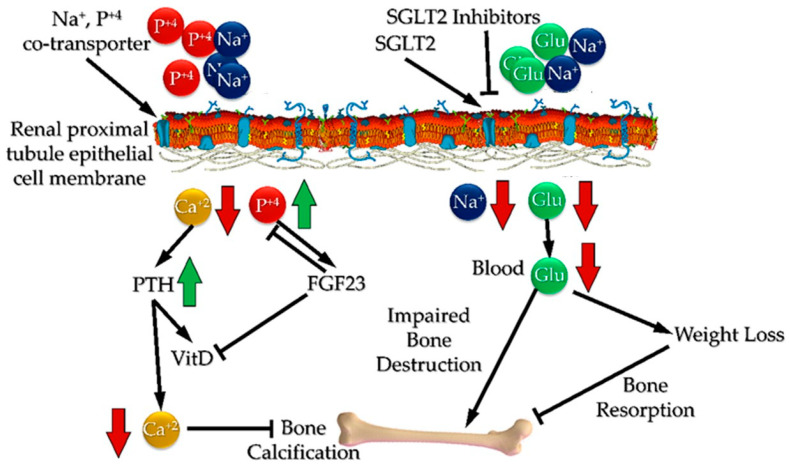
The potential mechanism of SGLT2 inhibitors on bone metabolism. SGLT2 inhibitors reduce glucose and potassium reabsorption in the kidney, which in turn enhances the absorption of phosphate, leading to exceeding calcium urine excretion. At the same time, an excess of phosphates activates FGF23, which induces phosphaturia and inhibits vitD production. This leads to decreased calcium intestinal absorption and bone calcification. At the same time, PTH production is enhanced in order to induce bone remodeling. On the other hand, weight loss caused by SGL2 inhibitors leads to decreased bone resorption and enhanced bone destruction (Adapted from Ye et al. (2018) [24] under the Creative Commons License CY BY 4.0 doi:10.3389/fphar.2018.01517, figure was completely restructured. Cell membrane was adopted from https://en.wikipedia.org/wiki/Fluid_mosaic_model, under the CC BY-SA 3.0 license. Accessed 30 March 2025) (legend: SGLT2 inhibitors: sodium–glucose co-transporter 2 inhibitors; FGF23: fibroblast growth factor 23; PTH: parathyroid hormone).Red arrows imply downregulation and green arrows imply upregulation.

There are several hypotheses regarding the mechanism of action of this class of drugs on bone metabolism. First of all is the direct effect on calcium homeostasis, because of their action on sodium–glucose co-transporters, resulting in an increase in parathyroid hormone (PTH) and decrease in 1,25 dixydroxy-vitamin D levels. Moreover, an indirect effect occurs through weight loss and by advancing glycation end products (AGEs), which in turn make bones more stiff [23] (Figure 3).

## 4. Insights into SGLT2 Inhibitors and Bone Metabolism

According to the literature, SGLT2 inhibitors are antidiabetic agents with multipotent effects, not only reducing hyperglycemia but also being beneficial in heart failure and chronic kidney disease (CKD). However, the debate still exists as to whether they could alter bone metabolism and increase the fracture incidence, or if they have a neutral effect.

Most studies agree that SGLT2 inhibitors and bone metabolism, including BMD and fractures, might have an interconnection, which, however, is yet to be elucidated. Hence, the majority of reports found no direct connection, with the exception of a research study by Koshizaka et al. (2021) [25], who found an increase in biomarkers of osteoclastogenesis. However, this has been reported in the cases of serial co-administration of ipragliflozin that was added to sitagliptin, where the tartrate-resistant acid phosphatase-5b (TRACP-5b) levels and bone mineral density did not exhibit any significant differences. This could be attributed to the fact that some patients were older, with possible co-morbidities (as for example, some included postmenopausal women), which might influence the outcome. In addition, in the aforementioned study, patients with osteoporosis or bone fractures were not included, which raises the question of efficacy in patients with osteoporosis. The BMD evaluation was performed with computer tomography (CT) and not with dual-energy X-ray absorptiometry (DXA) for the T-score calculation. This study was in agreement with a recent study by Blau et al. (2018) [26], who found that ganagliflozin has a positive effect on bone resorption markers, especially on FGF23 and PTH, although this was observed as a dose-dependent effect. However, this study also did not evaluate BMD and bone fractures as an endpoint. In addition, another very recent research study reported the beneficiary effects of empagliflozin on diabetic osteoporosis [27].

Another recent meta-analysis by Li et al. (2019) found that SGLT2 inhibitor treatment did not alter either BMD or the risk of fractures with respect to age, sex and HbA1c levels. In addition, this treatment did not affect the BMD at four skeletal sites [28]. These results were in agreement with a latter meta-analysis by Wang et al. (2023) [29], who found no changes in markers of bone metabolism and fracture incidence in diabetic patients.

Interestingly, a recent research study by Adimadhyam et al. (2019) [30] showed an increase in fracture incidence at the beginning of treatment in diabetic patients who received inhibitor treatment. Similarly, the same results were presented in another research study by Jackson et al. (2020) [31], yet this was only found to hold true in cases where ganagliflozin was used. Finally, these findings were in agreement with two recent meta-analyses by Li et al. (2019) [28] and Wang et al. (2023) [29], which did not find any significant effect of SGLT2 inhibitors, either on bone metabolism or on bone fractures.

These different outcomes among different studies could probably be attributed to the fact that the study populations were heterogeneous and the endpoint results were different. Also, another important issue reported in both research, as well as meta-analytic studies, is the duration of all projects/studies, which ranged between three and four months in total. An exception was the research study by Han et al. (2021) [32], which lasted for one year. Another possible reason for differences amongst studies could be attributed to the fact that the pathophysiology of osteoporosis differs among sexes. Thus, the results could be dependent upon the male/female distribution in a study cohort. Usually, studies did not discriminate between fracture incidences with respect to gender, and the presence or not of menopause was also usually not considered.

In addition, another significant aspect in several studies is the confounding effect of age. It is known that an individual’s bone mineral density reduces with age; thus, it is expected to be lower compared to younger cohorts. This also means that the fracture incidence could be due to the age factor and not due to treatment (meaning SGLT2 inhibitor treatments). In the present literature search, two research studies were found, by Haraguchi et al. (2020) [33] and Han et al. (2021) [32], which included older patients, yet with a small sample size. Another important factor in investigating the effects of SGLT2 inhibitors includes the fact that diabetes patients potentially have several co-morbidities, which might affect bone metabolism.

In another recent research study by Masajtis-Zagajewska et al. (2021) [34], patients with stage 3 kidney disease were included, but not with diabetes, which also reduces the bone mineral density. This study investigated bone metabolism biomarkers for a short period of time, which did not exhibit any relation to fragility. An interesting systematic review was conducted by Ye et al. (2018) indicating an increase in bone due to falls but not to SGLT2-inhibitors administration [24]. Yet, in a recent study, it was shown that treatment with dapagliflozin decreased FGF23, sclerostin and OPG levels, hinting towards an improvement in bone metabolism factors [35].

Several reports have highlighted that SGLT2 inhibitors had an enhancing effect on PTH and C-Terminal Telopeptide (CTX) levels, while at the same time down-regulating ALP levels [36]. This appeared to be controversial, since elevated PTH levels are related to bone formation, with a subsequent elevation in ALP levels. These findings added more complexity to the understanding of SGLT2 inhibitors and bone metabolism. Thus, it appears that this particular inhibitor acts or affects other signal transduction pathways, which are not yet well understood. Further on, it was reported that canagliflozin probably could decrease BMD, which, however, could be attributed to weight loss or the reduction in estradiol levels that is caused by this agent. It is known that weight loss, along with visceral fat reduction, could lead to an increase in CTX levels, which is a bone turnover marker, and thus could lead to a decrease in total hip BMD [25,37]. It is worth noting that since weight loss is tightly linked to the leptin/adiponectin ratio, a recent study showed that the administration of remogliflozin led to an increase in this ratio, which presented a potential role in bone metabolism [38]. As a result, it has been reported that the fracture risk could potentially increase after canagliflozin treatment, probably due to other co-morbidities. In most cases, patients were older, with a prior history and/or risk of cardiovascular disease, lower baseline estimated glomerular filtration rate and higher baseline diuretic use. In addition, the factor of accidental falls should be included in the equation, which leads to an increased fracture incidence, yet the exact relation and mechanism between the fracture risk and canagliflozin treatment still remains to be elucidated.

The literature search in the current study still did not reveal a clear effect on bone metabolism and fracture incidence with respect to the role of SGLT2 inhibitors. The main outcome of both research/experimental and meta-analytic studies was that there is still a controversy on SGLT2’s impact on bone metabolism, whereas only ganagliflozin showed an increased fracture incidence.

## 5. Discussion

In the present work, we attempted to review the literature concerning the impact of SGLT2-inhibitors on bone metabolism and especially on fracture incidence. It is already known that diabetes mellitus is associated with an increased incidence of bone fragility, where it is hypothesized that the presence of diabetes almost doubles the probability of fractures [39]. In addition, due to the complications of these patients, bone fractures increase morbidity and mortality. The underlying mechanism of diabetes mellitus and bone fractures has not yet clearly been clarified. Although there is a connection between some antidiabetic agents and bone metabolism, such as pioglitazone, currently, it is little understood for the novel category of SGLT2 inhibitors. It is noteworthy that there are not many studies directly addressing this topic. In other words, studies that investigate the effects of diabetes, along with SGLT2 inhibitors, on bone metabolism and fracture incidence, are sparse. Having said that, there is immense interest in understanding the mechanics underlying those two phenomena, i.e., diabetes and bone metabolism.

It is known that in bone metabolism, PTH regulates calcium metabolism. In case of low calcium levels, the PTH levels are elevated, which subsequently stimulates RANK:/RANK/OPG expression, and in turn, the osteoblastic machinery is stimulated [40,41]. As mentioned above, several reports highlighted a controversial role of SGLT2 inhibitor treatment, with increased PTH but low ALP levels. This suggests two main things; first, that SGLT2 inhibitors act on bone metabolism through additional pathways, which are still to be elucidated, and second, that several research variables should be included, such as renal and cardiac function, sarcopenia and low body weight, as well as advanced age. In addition, most studies agreed on the fact that SGLT2 inhibitors had no significant effect on P1NP, TRACP 5b, Vitamin-D and osteocalcin levels, which could be attributed to the study duration (usually three to four months) and to a possible indirect mechanism of SGLT2 inhibitors, which alters calcium and phosphate homeostasis in the kidneys [29,36].

A very significant aspect and probably a reason for why studies are not easy to conduct (i.e., for this particular topic of diabetes and bone metabolism) is the diversity of the study cohorts. Several factors can play a significant role, such as age, gender, co-morbidities, etc. For example, in a centralized study (The Singapore Chinese Study), which included a total of 63,257 individuals, including both males and females, aged between 45 and 74 years old, the fracture incidence was twice as high in female compared to male participants [42].

## 6. Conclusions

Patients with type 2 diabetes mellitus are at a high risk of alterations in their bone metabolism. Many of the available antidiabetic agents could interfere in this pathophysiology, and some of them might increase the fracture incidence. SGLT2 inhibitors are a novel class, with pleiotropic effects in many organs such as the kidneys and heart, although their effect on bone metabolism and fracture incidence is still unclear. Most studies have a common denominator; SGLT2 inhibitors probably act on bone metabolism, but through multiple signaling pathways, whose machinery still remains to be elucidated. This conclusion is due to the fact that most studies reported controversial results regarding the effects of SGLT2 on bone metabolism, yet factors such as age, co-morbidities, gender and, most importantly, the time/duration of treatments were not taken into account; these variables are crucial in the case of metabolic bone diseases such as osteoporosis. On the other hand, a more concise overview was derived for ganagliflozin, where the experimental evidence supported a probable negative effect on bone metabolism, giving it, therefore, a notable drug effect.

All studies agree on one thing, which is that further double-blind randomized controlled trials are required in order to understand SGLT2’s influence on bone metabolism and to ascertain whether this is a class or drug effect. Until we have more clinical data, all caregivers (medical and nursing staff) should be made aware of possible bone fractures in patients receiving this class of agents.

## Data Availability

Not applicable.

## Abbreviations

The following abbreviations are used in this manuscript:

BMD	Bone mineral density
CKD	Chronic kidney disease
CT	Computer tomography
DM	Diabetes mellitus
DXA	Dual-energy X-ray absorptiometry
FGF23	Fibroblast growth factor 23
GLP1RAs	Glucagon-Like Peptide-1 Receptor Agonists
IDF	International Diabetes Federation
IFNγ	Interferon gamma
M-CSF	Macrophage colony-stimulating factor
OPG	Osteoprotegerin
PDGF	Platelet-Derived Growth Factor
PPARγ	Peroxisome proliferator-activated receptor γ
PTH	Parathormone
RANKL	Receptor activator of nuclear factor-κB ligand
SGLT2	Sodium–glucose co-transporter 2
T2DM	Type 2 diabetes mellitus
T4	Thyroxine
TNF	Tumor Necrosis Factor
TRACP-5b	Tartrate-resistant acid phosphatase-5b
TZD	Thiazolidinedione
Vit D	Vitamin D
WHO	World Health Organization
